# Fatigue Life Prediction of Pavement Base Layers Using Supersulfated Cement-Treated Aggregates Considering Stress-Dependent Resilient Modulus

**DOI:** 10.3390/ma19142952

**Published:** 2026-07-09

**Authors:** Jianying Deng, Xingyu Hu, Yucheng Li, Tiqiang Shan, Yuqing Zhang, Yang Zhou

**Affiliations:** 1 Shandong Hi-Speed Infrastructure Construction Co., Ltd., Jinan 250000, China; 2 School of Transportation, Southeast University, Nanjing 211189, Chinazhangyuqing@seu.edu.cn (Y.Z.); 3 School of Materials Science and Engineering, Southeast University, Nanjing 211189, China

**Keywords:** super-sulphated cement treated aggregates, resilient modulus, stress non-linearity, fatigue cracking, finite element method

## Abstract

To reduce carbon emissions from cement-treated aggregate base layers and examine the nonlinear service behavior of semi-rigid materials, supersulfated cement (SSC) was used to replace ordinary Portland cement (OPC). A dynamic triaxial loading protocol was adopted to separate the effects of bulk stress and shear stress on the dynamic resilient modulus of supersulfated cement-treated aggregate (SSC-CTA). A fatigue damage equation was developed based on the strain energy balance during cracking, and Paris’ law damage parameters were introduced to compare the damage growth rates of SSC-CTA and ordinary Portland cement-treated aggregate (OPC-CTA). Finite element analysis and partial differential equations were further used to link the stress-dependent resilient modulus with structural fatigue life. The results show that SSC-CTA had a lower dynamic resilient modulus than OPC-CTA under the same stress state. The average resilient modulus of SSC-CTA was 978 MPa, which was 15.47% lower than that of OPC-CTA. For both materials, the modulus increased with bulk stress and decreased with octahedral shear stress, and the NCHRP 28A model accurately predicted this nonlinear behavior. Although SSC-CTA had a lower modulus, its indirect tensile strength reached 864.3 kPa, representing a 52.65% increase compared with OPC-CTA. The Paris’ law parameters further indicated that SSC reduced the damage growth rate during crack propagation. The finite element results showed that the predicted structural fatigue life of SSC-CTA increased by 4.49–35.90% under different load levels.

## 1. Introduction

Supersulfated cement (SSC) is a green binder mainly composed of industrial by-products, such as slag and gypsum [1]. The content of added Portland cement is usually less than 5%, giving it lower carbon emissions than ordinary Portland cement (OPC). Therefore, it has been widely used in concrete materials [2,3]. In addition, the slight expansion of SSC can reduce early-age microcracks caused by drying shrinkage and thermal shrinkage [4]. Its application has therefore been extended to cement-treated aggregate base materials for road pavements. Common road base materials include foamed asphalt-treated aggregate and Portland cement-treated aggregate. Although these materials have good structural integrity, they still show stress-dependent nonlinear behavior in laboratory tests [5]. Supersulfated cement-treated aggregate (SSC-CTA) has better cracking resistance and carbon-reduction potential [6]. However, the weakened cementation of the binder may make its nonlinear behavior more pronounced. Specifically, its resilient modulus may show strong stress dependence [7,8], which further affects the fatigue life of the whole base layer.

Many specifications and design systems classify chemically stabilized aggregate layers as elastic materials and use the elastic or resilient modulus at a specified curing age as the design input. For example, China’s JTG D50 uses the modulus of cement-stabilized materials at 90 days [9], while American Association of State Highway and Transportation Officials (AASHTO)-based pavement design [10] and ASTM-based laboratory testing procedures also rely on modulus- or strength-related parameters of cement-treated materials [11]. However, many experimental studies have shown that cement-stabilized materials have nonlinear modulus behavior. Their resilient modulus is affected by the loading level. For example, Zhang et al. [12] used a UTM testing system to measure the dynamic and static resilient moduli of semi-rigid base materials with three cement contents under different loading modes. The results showed that semi-rigid base materials have different stress-dependent behavior under compression and flexural-tensile loading. As the stress level increased, the compressive modulus gradually increased, whereas the flexural-tensile modulus gradually decreased. Wang et al. [13] found that semi-rigid base materials show obvious nonlinear stress–strain behavior under certain loading conditions. They established stress–strength correlation models for semi-rigid materials under shear and flexural-tensile loading using quadratic and power functions, respectively. Based on extensive dynamic resilient modulus tests on cement-stabilized lateritic aggregates, Shi [14] introduced a Boltzmann function with an S-shaped curve, similar to that used for asphalt mixtures. A modulus model considering the coupled effects of loading frequency and stress was then developed. In practice, pavement materials are usually subjected to three-dimensional stress states under traffic loads and environmental conditions. A single axial loading mode ignores the effect of confining pressure on the resilient modulus. Therefore, for some weakly cemented mixtures, researchers have evaluated the resilient modulus of inorganic binder-stabilized materials by referring to the dynamic triaxial tests for granular and subgrade materials in Chinese asphalt pavement design specifications or AASHTO standards. Luo et al. [15] adopted the Uzan model to predict the resilient modulus of cement-stabilized materials. The results showed that the resilient modulus increased under higher confining pressure. Based on AASHTO, Su [16] proposed a loading protocol with a higher maximum axial load to represent the high stress states that may occur in semi-rigid bases during service. Laboratory triaxial tests showed that the resilient modulus of cement-stabilized bases was highly dependent on deviator stress but was not sensitive to confining pressure. Mohammadinia et al. [17] compared the performance of cement-treated recycled asphalt mixture, recycled concrete aggregate, and crushed brick by conducting repeated-load triaxial tests on different aggregates. The results showed that the resilient modulus of different aggregates increased with cement content, curing time, and confining pressure.

In addition, the damage variable is an important index for describing the evolution of fatigue damage. Fatigue damage can be defined in different ways, but a unified evaluation criterion has not yet been established [18]. Early studies defined fatigue damage from a macroscopic and phenomenological perspective. The damage variable was often expressed as the ratio of the current number of load cycles to the fatigue life or the reduction ratio of modulus or strength [19,20]. However, Fatigue damage is essentially caused by the long-term action of repeated loading. It involves relative movement within the internal microstructure and the growth of microcracks from initial defects, which further leads to the degradation of macroscopic mechanical properties [21]. The decrease in modulus may result from several factors. In addition to fatigue cracking, the modulus of CTA can also change or decrease due to stress state and moisture condition [22]. Fatigue cracking is a physical process involving the initiation and evolution of microcracks under external cyclic loading. Current studies still lack a quantitative index that can directly describe and evaluate cracking. Therefore, when evaluating the fatigue performance of SSC-CTA, it is essential to establish a fatigue damage index directly related to cracking.

Although stress-dependent resilient modulus models have been widely used for granular and subgrade materials, their application to SSC-CTA remains limited. Moreover, existing studies on CTA usually evaluate resilient modulus, fatigue damage, and pavement structural response separately. In this study, the stress-dependent resilient modulus of SSC-CTA was first characterized by a dynamic triaxial loading protocol that separates the effects of bulk stress and octahedral shear stress. The fatigue damage growth was then quantified using a strain-energy-based damage equation combined with Paris’ law parameters. Finally, the laboratory-determined nonlinear modulus was incorporated into a finite element pavement model to evaluate its influence on structural fatigue life, thereby linking material properties with the mechanical response of the pavement structure.

## 2. Materials and Methods

### 2.1. Basic Materials

This study used P.O. 42.5 ordinary Portland cement to prepare OPC-CTA under conventional conditions. SSC-CTA specimens were prepared by replacing ordinary Portland cement with supersulfated cement. The supersulfated cement was blended at a mass ratio of slag: desulfurized gypsum: ordinary Portland cement = 16:3:1. In this ternary slag–sulfate–alkaline-component system, P.O. 42.5 ordinary Portland cement was used as the low-dosage alkaline component rather than as a conventional strong alkaline activator. The ordinary Portland cement mainly hydrates first and releases Ca(OH)_2_ and OH^−^, providing a mild alkaline environment for the dissolution of the slag glass phase. The desulfurized gypsum supplies SO_4_^2−^ and Ca^2+^, while the slag provides reactive Ca–Si–Al components. Therefore, the hardening process of SSC-CTA is mainly governed by hydration reactions, leading primarily to the formation of C-(A)-S-H gel and ettringite (AFt). The main component of desulfurized gypsum is CaSO_4_·1/2H_2_O. The slag used in this study was granulated blast furnace slag, which is a highly fine and reactive powder. The chemical compositions of P.O. 42.5 cement, granulated blast-furnace slag, and desulfurized gypsum are listed in Table 1. Limestone aggregates from Jinhua, Zhejiang Province, were selected for the tests.

### 2.2. Specimen Preparation

For inorganic binder-stabilized materials, the mixture proportion and optimum moisture content are important factors affecting their mechanical properties. In this study, the aggregate gradation was designed according to the recommended range in the Technical Guidelines for Construction of Highway Roadbases (JTG/T F20-2015) [23], as shown in Table 2.

**Figure 1:**
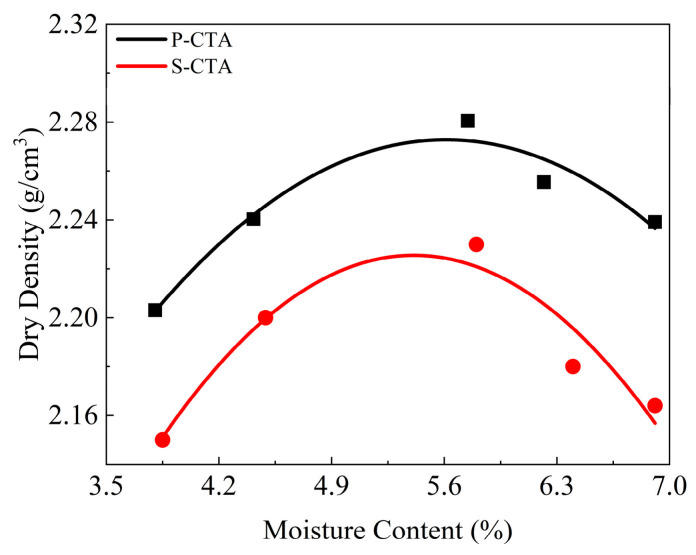
Dry density–moisture content curves of CTA.

The maximum dry density (MDD) and optimum moisture content (OMC) were determined by compaction tests according to the Test Methods of Materials Stabilized with Inorganic Binders for Highway Engineering (JTG 3441-2024) [24]. The density–moisture content curves of the specimens with a binder content of 4.0% are shown in Figure 1. The OMC and MDD of OPC-CTA and SSC-CTA were 5.6% and 2.27 g/cm^3^, and 5.41% and 2.22 g/cm^3^, respectively. After the mixture design test, the quantities of different materials were calculated according to the specification. Both OPC-CTA and SSC-CTA were prepared with a binder content of 4.0% by mass of dry aggregate. The maximum particle size used in this study was no greater than 19 mm, which belongs to medium-sized inorganic binder-stabilized materials. Therefore, cylindrical specimens with a diameter and height of 100 mm × 150 mm were prepared for the dynamic triaxial resilient modulus test to reduce the influence of boundary effects. For the indirect tensile fatigue test, the specimen thickness is generally required to be more than three times the maximum particle size. Therefore, disc specimens with a diameter and thickness of 100 mm × 60 mm were used. After demolding, all specimens were cured for 28 days in a standard curing chamber at 20 °C and 95% relative humidity.

### 2.3. Laboratory Test Methods

1. Dynamic Triaxial Loading Test

The current Test Methods of Materials Stabilized with Inorganic Binders for Highway Engineering only recommends a testing method for the compressive resilient modulus of CTA under uniaxial loading. However, during actual service, the base layer is subjected to multidimensional forces from traffic loads and the confining pressure provided by surrounding materials. A single axial loading method may therefore fail to obtain the actual modulus of the material. In this study, the triaxial resilient modulus tests for granular and subgrade materials specified in AASHTO T307-99 and the Specifications for Design of Highway Asphalt Pavements were adopted as references [9,10]. A loading protocol was designed, as shown in Table 3. In this protocol, the bulk stress and octahedral shear stress were kept constant in every three loading sequences to investigate the effects of different stress modes on the resilient modulus. In addition, the stress ranges of the cyclic axial load and confining pressure were expanded to meet the stiffness sensitivity requirements of CTA. Sequence 0 was used as a conditioning stage before the formal resilient modulus measurement. The 1000 loading cycles were applied to stabilize the contact between the specimen and the loading system and to reduce the influence of initial seating and compaction on the subsequent measured resilient modulus.

2. Indirect tensile strength test

The indirect tensile strength test was conducted not only to compare the tensile performance of OPC-CTA and SSC-CTA, but also to determine the initial fatigue load for the subsequent indirect tensile fatigue test and provide parameters for surface energy calculation. A monotonic load was applied using a multifunctional hydraulic servo dynamic testing system for pavement materials (DTS-30), with a loading rate of 1 mm/min.

3. Indirect tensile fatigue test

The same type of specimens used in the indirect tensile strength test was adopted for the indirect tensile fatigue test. Fatigue loading was applied using DTS-30. Considering the slow self-healing capacity of CTA after 28 days of curing, a continuous sinusoidal wave without rest periods was used, with a loading frequency of 10 Hz. The contact force was set to 10 N, and the cyclic axial force was taken as 0.8 times the peak load obtained from the indirect tensile strength test.

For each material type, parallel specimens were prepared and tested to ensure the repeatability of the experimental results. The reported values of indirect tensile strength were calculated as the average values of the parallel specimens. For the dynamic triaxial resilient modulus test, the resilient modulus was calculated within each loading sequence after the conditioning stage, and the coefficient of variation was used to evaluate data repeatability. The coefficient of variation of the resilient modulus within each loading sequence was lower than 2%, indicating good stability of the measured data. For model fitting, the coefficient of determination, *p*-value, and cumulative error were used to evaluate the reliability of the regression results.

### 2.4. Pavement Structure Modeling in FEM

**Figure 2:**
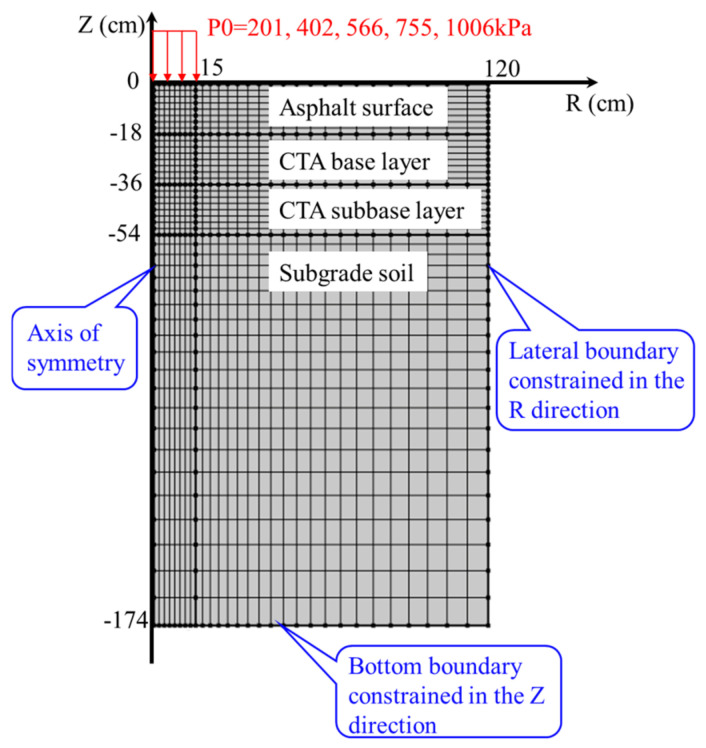
Finite element mesh of the pavement structure.

A two-dimensional axisymmetric finite element model of the pavement structure was established, as shown in Figure 2. Since the vehicle wheel load can be approximately regarded as acting over a circular area, and the pavement structure shows good geometric and loading symmetry on both sides of the wheel-load centerline, the model was simplified as a two-dimensional axisymmetric model. The wheel-load centerline was taken as the axis of symmetry, and an R–Z coordinate system was established. The R direction represents the radial horizontal distance, while the Z direction represents the vertical depth. The radial calculation range of the model was 1.2 m, and the vertical depth was 1.74 m. This model size can cover the main stress diffusion region in the pavement structure under vehicle loading and reduce the influence of boundary conditions on the mechanical response of the base layer. The pavement structure was divided into four layers from top to bottom: asphalt surface layer, CTA base layer, CTA subbase layer, and subgrade soil. The thicknesses of the asphalt surface layer, CTA base layer, and CTA subbase layer were all 18 cm. The subgrade soil extended to the bottom of the model. Continuous contact was assumed between adjacent layers. In other words, no relative slip or separation was considered at the layer interfaces, and displacement compatibility was satisfied. The boundary conditions were set according to the requirements of axisymmetric finite element analysis. The left boundary was defined as the axis of symmetry, and its radial displacement was constrained to ensure axisymmetric deformation. The right boundary was constrained in the R direction to limit horizontal deformation at the outer boundary. The bottom boundary was constrained in the Z direction to simulate the vertical support provided by the deep subgrade. The top surface of the model was set as a free boundary except for the loading area, and no additional displacement constraint was applied. The vehicle load was applied as a uniformly distributed vertical pressure over a circular loading area on the pavement surface. The loading radius was 15 cm. To investigate the mechanical response of the pavement structure under different load levels, five vertical pressures of 201, 402, 566, 755, and 1006 kPa were applied.

## 3. Results and Discussion

### 3.1. Investigation of the Stress Dependence of the Resilient Modulus of CTA

#### Dynamic Triaxial Resilient Modulus Test Results

**Figure 3:**
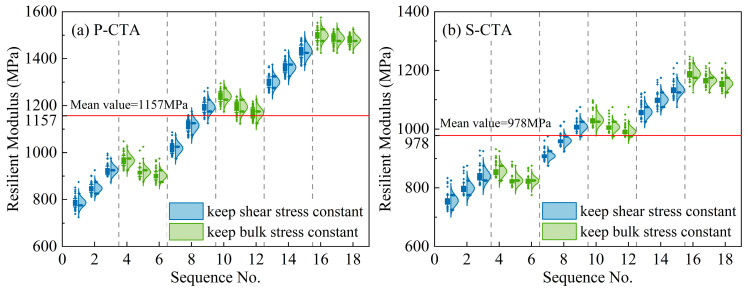
Resilient modulus results of CTA at a 28-day curing age.

In the dynamic triaxial resilient modulus test, the modulus was calculated using Equation (1). During each 100-cycle loading sequence, the coefficient of variation of the resilient modulus for different CTA mixtures was lower than 2%, confirming the reliability of the test data. Figure 3 shows the variation in resilient modulus of OPC-CTA and SSC-CTA after 28 days of curing under the loading sequences listed in Table 3. Under the separated bulk stress-shear stress loading condition, the resilient modulus curves showed a three-peak pattern with a stepwise increase as the loading sequence progressed. This indicates that the modulus is positively correlated with bulk stress but negatively correlated with shear stress. This behavior can be explained by the interaction between particles at the mesoscopic scale. Under shear stress, the tensile stress between particles increases, which reduces the frictional resistance and leads to a decrease in modulus. Under bulk stress, more compressive contacts are formed between aggregate particles, enhancing interlocking and friction and thereby increasing the modulus [8,25]. When shear stress *τ* was used as the independent variable and bulk stress *θ* was kept constant, the resilient modulus *Mr* decreased with increasing *τ*. In contrast, when *θ* was used as the independent variable and *τ* was kept constant, *Mr* increased with increasing *θ*. For OPC-CTA, the resilient modulus ranged from 700 to 1600 MPa over the full loading sequence, with an average value of 1157 MPa. Due to the lower cementation capacity of SSC, the resilient modulus of SSC-CTA ranged from 700 to 1200 MPa, and its average value decreased to 978 MPa, representing a reduction of approximately 15.47%. However, the minimum resilient modulus of SSC-CTA was close to that of OPC-CTA. This suggests that SSC-CTA can retain the load-bearing capacity of a semi-rigid base while showing cracking resistance comparable to that of a flexible base.

(1) $$M_{r}=\frac{\sigma_{\mathrm{cyclic}}}{\varepsilon_{r}}$$

where $\sigma_{\mathrm{cyclic}}$ is the cyclic stress, $\sigma_{\mathrm{cyclic}}=\sigma_{d}$; $\varepsilon_{r}$ is the resilient strain.

**Figure 4:**
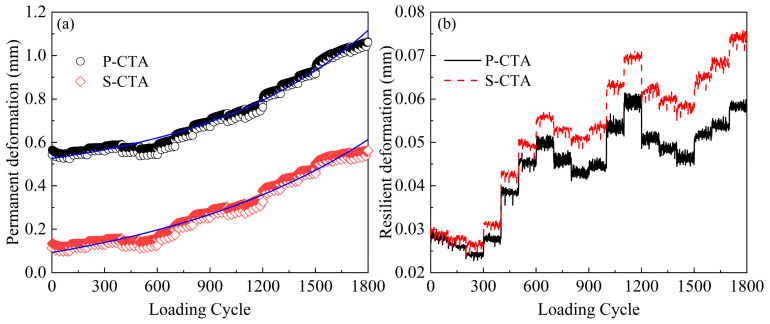
Deformation trends of CTA at a 28-day curing age. (**a**) Permanent deformation accumulation; (**b**) Resilient deformation evolution.

Figure 4a,b shows the variations in permanent deformation and recoverable deformation of OPC-CTA and SSC-CTA during loading. The results indicate that CTA exhibits clear elastoplastic constitutive behavior. Under the loading levels used in this study, the permanent deformation of the material can reach 3–10 times the recoverable deformation. The plastic strain caused by permanent deformation accumulates continuously with increasing loading cycles and loading levels, and its final growth rate follows an exponential pattern. Under the same loading mode, SSC-CTA showed lower permanent deformation and higher recoverable deformation than OPC-CTA. This is because SSC can compensate for shrinkage throughout the hydration process, greatly reducing the initial cracks caused by early drying shrinkage and thermal shrinkage [26]. As a result, there is less space for particle movement under loading, which reduces permanent deformation [27]. OPC-CTA forms a high-stiffness but brittle network due to its strong cementation. It therefore has a higher resilient modulus but is more prone to failure caused by microcrack propagation [28]. In contrast, SSC-CTA behaves more like a flexible material. It has a lower resilient modulus, while its internal pores are better filled.

To investigate the effects of different stress types on CTA and to accurately predict its resilient modulus, two prediction models, as expressed in Equations (2) and (3), were used to analyze the measured resilient modulus. Equation (2) is the two-parameter Uzan model, which mainly considers the effect of deviator stress on the resilient modulus. Equation (3) is the three-parameter NCHRP 28A model, which considers the combined effects of bulk stress and octahedral shear stress.

(2) $$M_{r}=k_{1}P_{a}{\left(\frac{\sigma_{d}}{\mathrm{Pa}}\right)}^{k_{2}}$$

(3) $$M_{r}=k_{3}P_{a}{\left(\frac{\theta }{\mathrm{Pa}}\right)}^{k_{4}}{\left(\frac{\tau_{\mathrm{oct}}}{\mathrm{Pa}}+1\right)}^{k_{5}}$$

where $\sigma_{d}$ is the deviator stress; $k_{1}$, $k_{2}$, $k_{3}$, $k_{4}$ and $k_{5}$ are regression parameters; and Pa is the atmospheric pressure, taken as 0.1013 MPa.

Multiple linear regression and Solver-based optimization were used to fit and predict the measured resilient modulus. The regression parameters, coefficient of determination (R^2^), *p*-value, and cumulative error were then determined for each model. The cumulative error was calculated using Equation (4). Table 4 presents the regression results of the resilient modulus of OPC-CTA and SSC-CTA under different models.

(4) $$\Delta_{\mathrm{cul}}=\frac{\left|Mr_{\mathrm{measured}}-Mr_{\mathrm{predict}}\right|}{Mr_{\mathrm{measured}}}$$

where $\Delta_{\mathrm{cul}}$ is the cumulative error, and $Mr_{\mathrm{measured}}$ and $Mr_{\mathrm{predict}}$ are the measured and predicted resilient moduli, respectively.

Under the NCHRP 28A model, the coefficients of determination for both materials reached 0.99, and the cumulative errors were lower than 25%. This indicates that the NCHRP 28A model has higher prediction accuracy than the Uzan model. In terms of model parameters, $k_{1}$, $k_{2}$, $k_{3}$, and $k_{4}$ were always greater than 0, while $k_{5}$ was less than 0. This indicates that bulk stress and deviator stress promote the resilient modulus of CTA with different binders, whereas octahedral shear stress reduces the modulus. Except for $k_{1}$, the absolute values of the parameters of SSC-CTA cured for 28 days were lower than those of OPC-CTA. Combined with the permanent and recoverable deformation results, this further indicates that SSC-CTA has a stronger ability to resist deformation caused by external loading.

### 3.2. Investigation of the Fatigue Performance of CTA

#### 3.2.1. Determination of Fatigue Load

The indirect tensile strength of CTA with different binders was measured. The average value of parallel specimens was taken as the indirect tensile strength. The fatigue test load was then determined according to the stress ratio, as listed in Table 5. Under the same curing conditions, SSC-CTA cured for 28 d showed better tensile performance. Its indirect tensile strength increased by 52.65% compared with that of OPC-CTA. In addition to the indirect tensile strength and fatigue stress, Table 5 also reports the material input parameters used in the subsequent fatigue damage model, including the initial resilient modulus and air void content. The air void content was obtained from previous CT tests on CTA specimens prepared under comparable mixture proportions and compaction conditions [7].

The indirect tensile strength test was used to determine the fatigue loading level, while the fatigue test setup with side-mounted LVDTs was used to record the recoverable and permanent horizontal deformation during cyclic loading. During the indirect tensile fatigue test, metal plates used to fix the LVDTs were attached to the symmetric outer surfaces of the cylindrical specimen using epoxy resin. During installation, the horizontal reference plane was aligned with the central axis of the specimen, and the longitudinal centerline of each metal plate was kept parallel to the specimen axis. The LVDTs on both sides were used to measure the recoverable deformation, permanent strain, and maximum horizontal deformation of the specimen. These measurements were then used to calculate the horizontal resilient modulus. The program automatically recorded the modulus change caused by fatigue loading during the test. The fatigue loading termination condition was determined based on the initial horizontal resilient modulus. In this study, the material was considered to have reached the fatigue limit when the modulus decreased to 98% of its initial value or when specimen failure occurred.

#### 3.2.2. Establishment of the Fatigue Damage Model

Based on the energy balance principle of continuum damage mechanics and the assumption that the energy stored in a damaged material is equal to that stored in the corresponding undamaged material, the damage density was defined as the ratio of the crack area in the local cracking zone to the total area [31], as expressed in Equation (5).

(5) $$\xi =\frac{S^{C}}{S^{A}}=\frac{c}{d}$$

where *ξ* is the damage density; $S^{C}$ and $S^{A}$ are the area of the local cracking zone and the actual area of the specimen under indirect tensile loading, respectively. Here, $S^{C}$ = *ch*, where *c* is the crack length and *h* is the specimen thickness of 60 mm. $S^{A}$ = *dh*, where *d* is the specimen diameter of 100 mm.

Based on the strain energy balance principle of Griffith fracture theory, an energy balance equation was further established [32]. The total strain energy stored in the apparent configuration is equal to the sum of the total strain energy stored in the actual configuration and the increased surface energy on the crack surfaces, minus the strain energy released by crack propagation. Thus, a cubic equation with respect to the damage density can be obtained [33]:

(6) $$a_{1}\xi^{3}+a_{2}\xi^{2}+a_{3}\xi +a_{4}=0$$

where $a_{1}=2\Gamma$, $\Gamma =\frac{\sigma_{H}^{2}\pi c}{E_{0}}$ is the surface strain energy of the material (N/m); *c* is the initial crack length, and $c=0.0037(a_{v}{)}^{2}+0.0071\left(a_{v}\right)+0.5583$; *a_v_* is air void content in percentage; $\sigma_{H}$ is the horizontal stress, which equals to fatigue stress; in this study, Γ was taken as 0.047 N/m for OPC-CTA and 0.060 N/m for SSC-CTA, respectively; $a_{2}=-4\Gamma -\frac{{\sigma_{H}}^{2}\alpha d}{2E_{r}}$ and $\alpha =\frac{w}{d}$, w is the width of the local cracking zone, which is approximately equal to the width of the loading strip and is taken as 19 mm; *d* is the diameter of specimen; $E_{r}$ is the horizontal resilient modulus of the specimen at each loading cycle; $a_{3}=2\Gamma +\frac{{\sigma_{H}}^{2}\alpha d}{E_{r}}-\frac{{\sigma_{H}}^{2}\alpha d}{4E_{0}}$, $E_{0}$ is the initial horizontal resilient modulus of the specimen; $a_{4}=\frac{{\sigma_{H}}^{2}\alpha d}{2E_{0}}-\frac{{\sigma_{H}}^{2}\alpha d}{2E_{r}}$.

**Figure 5:**
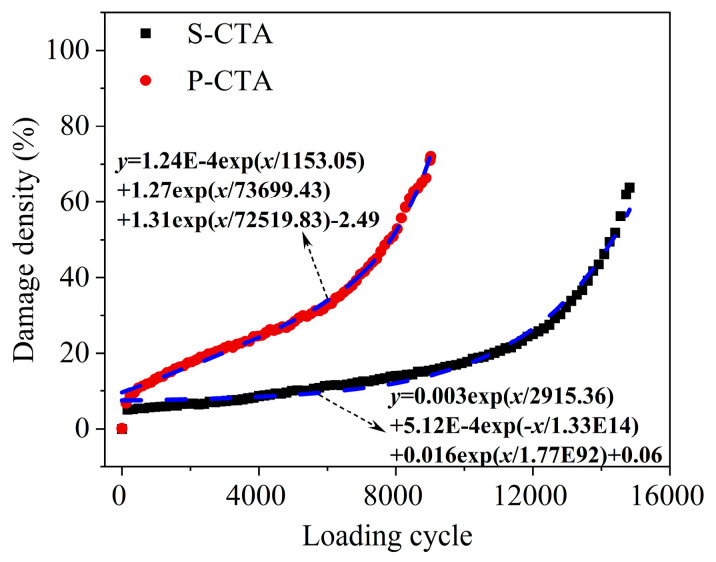
Variation of damage density with cyclic loading for CTA.

Figure 5 shows the relationship between damage density *ξ* and the number of fatigue loading cycles under different conditions. To obtain the coefficients related to Paris’ law and determine the damage growth rate, Equation (7) was used to fit the curves. The damage density of both CTA mixtures increased at an accelerating rate with the number of loading cycles. For SSC-CTA, although a higher indirect tensile load of 700 kPa was applied, more loading cycles were required to reach the same damage density. This indicates that the incorporation of SSC improves the cracking resistance of CTA. This improvement can be attributed to the slight expansion of SSC, which reduces the initial microcracks [34]. The expansion stress can also disperse the tensile stress to some extent, thereby inhibiting crack propagation.

(7) $$\xi =A^{\prime }\exp(B\cdot N)+C\exp(D\cdot N)+E\exp(F\cdot N)+G$$

where $A^{\prime },\;B,\;C,D,E,F$ and are regression parameters; *N* is the cyclic number.

After obtaining the damage density of the specimens, Paris’ law was used to characterize the damage growth rate of the material under cyclic loading, as expressed in Equation (8):

(8) $$\frac{d\xi }{dN}=A{\left(\Delta J\right)}^{n}$$

where *A* and *n* are the model coefficients of Paris’ law; ΔJ is the J-integral.

The incremental J-integral is equivalent to the dissipated work per unit newly created crack surface area, as expressed in Equation (9):

(9) $$\Delta J=\frac{\partial \left(DSE\cdot V\right)}{\partial \left(2S^{A}\xi \right)}\approx \frac{h}{2}\frac{\partial }{\partial \xi }\left(\frac{\sigma_{H}^{2}}{E^{\prime }}-\frac{\sigma_{H}^{2}}{2E_{r}}\right)$$

where DSE is the dissipated strain energy density for crack growth, and DSE is the energy (density) which is dissipated solely for crack growth, which excludes the energy dissipation for viscoelastic relaxation; V is the volume of the laboratory sample and h is the sample height; $E^{\prime }=E_{0}{\left(1-\xi \right)}^{2}$ and $E_{r}=E_{0}\left(1-\xi \right)$ are defined as secant modulus and recovery modulus; $2S^{A}\xi$ computes the total area of the cracks in the sample. Substituting all parameters into Equation (9) gives $\Delta J=\frac{h}{2}\frac{\sigma_{H}^{2}}{2E_{0}}\frac{\left(3+\xi \right)}{{\left(1-\xi \right)}^{3}}$.

**Figure 6:**
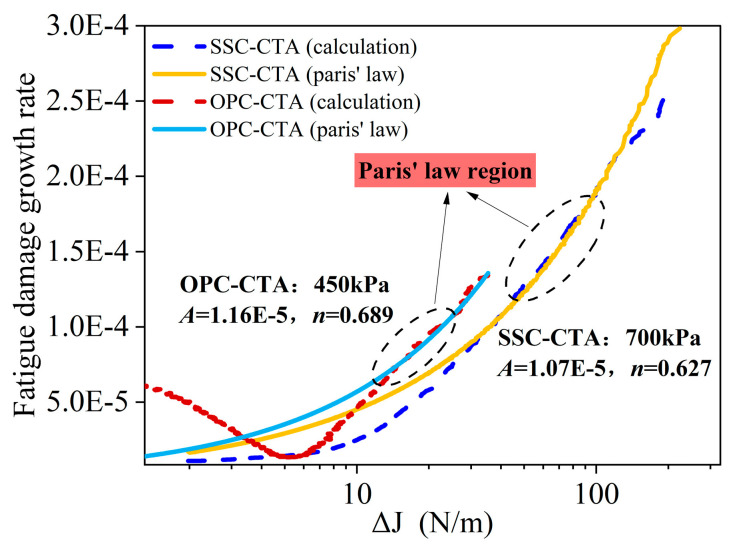
Fatigue crack growth rate of CTA.

After obtaining the damage density of the specimens, Paris’ law was used to characterize the relationship between the fatigue damage growth rate and the ΔJ, as expressed in Equation (9). To clarify the applicability of this relationship, Figure 6 presents the calculated damage growth rate as a function of ΔJ, together with the corresponding Paris’ law fitting curves for OPC-CTA and SSC-CTA. At low ΔJ levels, the calculated damage growth rates of both materials deviate from the Paris’ law fitting curves. This indicates that the early stage of fatigue loading is not fully controlled by stable crack propagation, but is associated with crack initiation, damage adaptation, and local adjustment of the internal structure. In this stage, SSC-CTA shows a lower damage growth rate than OPC-CTA even under a higher applied fatigue stress, suggesting stronger resistance to early microcrack development. This behavior can be attributed to the slight expansion effect of SSC, which helps reduce early drying-shrinkage and thermal-shrinkage microcracks [35]. As ΔJ increases, the calculated damage growth rates gradually become more consistent with the Paris’ law fitting curves, especially within the highlighted Paris’ law region. This indicates that the Paris-type power-law relationship is more suitable for describing the main crack propagation stage rather than the entire fatigue damage process. For OPC-CTA, the transition into the Paris’ law region is more pronounced, indicating a clearer acceleration of crack propagation. In contrast, SSC-CTA shows a more gradual increase in damage growth rate with increasing ΔJ, suggesting improved resistance to fatigue crack propagation. The A and *n* values obtained from the Paris’ law model were 1.07 × 10^−5^ and 0.627, respectively, which were lower than those of OPC-CTA. This further confirms that the slight expansion of SSC improves the cracking resistance of CTA.

### 3.3. Fatigue Life Prediction of Pavement Structure

#### 3.3.1. Description of Finite Element Modeling Parameters

Table 6 summarizes the pavement structure used in the model, including the thickness of each layer, constitutive model, and input parameters for finite element analysis. To investigate the evolution of the structural response of semi-rigid base layers with different binders, the laboratory dynamic triaxial resilient modulus test results of SSC-CTA and OPC-CTA were used as input parameters for structural design. A nonlinear viscoelastic constitutive model was adopted for the asphalt pavement layers, while an elastic constitutive model was used for the subgrade soil [36].

Table 7 summarizes the input parameters of each structural layer. To simplify the simulation, Poisson’s ratio was assumed to be constant, and the same set of parameters was used for the base and subbase layers. For the CTA base layer, the nonlinear elastic model was described by the NCHRP 28A model, which was used to consider the stress dependence of cement-stabilized materials. The values of *k*_1_–*k*_3_ were taken from the results presented above. The initial modulus was determined from the minimum modulus measured in the repeated-load triaxial test. According to the difference between the modulus values obtained by the top-surface method and the side-surface method in the specification, the value of k_1_ was uniformly increased by three times to reduce the modulus deviation caused by artificial strain in the top-mounted LVDT test setup. For the asphalt mixture layer, a solid-like generalized Maxwell model, as expressed in Equation (10), was used to characterize the relaxation modulus of the material. The dynamic modulus test was conducted strictly according to the ASTM standard.

(10) $$E\left(t\right)=E_{\infty }+\sum_{i=1}^{M}E_{i}\exp\left(-\frac{t}{\tau_{i}}\right)$$

where *t* is time, and *M* is the total number of Maxwell elements. In this study, *M* = 11.

The pavement structure was modeled using COMSOL Multiphysics 6.2. Its built-in viscoelastic model can accurately describe the constitutive behavior of asphalt mixtures. For the CTA base layer, the finite element modeling method based on the weak-form equation proposed by Zhang et al. [37] was adopted to simulate the nonlinear stress-dependent behavior. The model was defined in the finite element system as follows:

(11) $$\{\begin{array}{l} -\left(u11-\mathrm{solid}.I1s\right)*test\left(u11\right)\\ -\left(u12-sqrt\left(\mathrm{solid}.II2s*2/3\right)\right)*test\left(u12\right) \end{array}$$

where solid.I1s and solid.II2s represent the first invariant of the stress tensor (*I*_1_) and the second invariant of the deviatoric stress tensor (*J*_2_), respectively. Both variables are predefined in COMSOL and can be directly used in the weak-form expression. *u*11 and *u*12 are dependent variables defined in COMSOL, with units of N/m^2^.

The stress-dependent nonlinear equation of CTA was further defined in COMSOL as follows:

(12) {Mr=k1∗Pa∗(abs(u11)/Pa)^k2∗(u12/Pa+1)^k3Ea=if(Mr>Em,Mr,Em)Ez=if(u11<0,Ea,Em)

where $E_{a}$ is a transition variable defined in COMSOL, and $E_{z}$ is the modulus assigned to the base layer.

Based on Equation (8), the remaining fatigue life of the material can be further defined as:

(13) $$N_{f}=\int_{0}^{\xi }A^{-1}{\left(\Delta J\right)}^{-n}d\xi$$

To numerically predict the fatigue life in the finite element model, the expanded expression of Δ*J* was substituted into the fatigue life equation. A corresponding user-defined numerical integration expression was then established in the global definitions of COMSOL:

(14) $$Integrate((1/A)*((solid.sr{)}^{2}/(2*E_{z})*H/2*(3+x)/(1-x{)}^{3}{)}^{-n},x,0,\xi^{*})$$

where *Integrate*(…, *x*, 0, ξ*) denotes definite integration with damage density as the integration variable over the interval [0, ξ*]. solid.sr is the horizontal stress in the base layer, *H* is the thickness of the base layer, and ξ* is the damage factor. In this study, fatigue failure was assumed to occur when ξ* = 0.9.

#### 3.3.2. FEM Test Results

**Figure 7:**
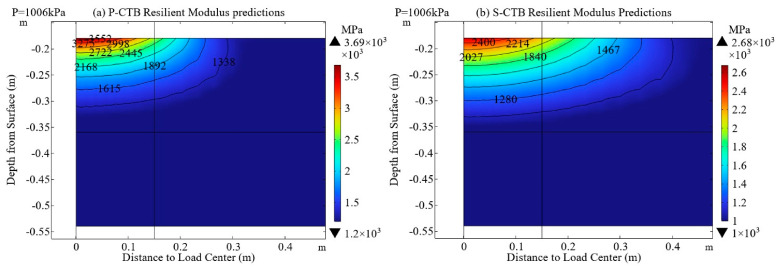
Distribution of stress-dependent resilient modulus for two base types.

Figure 7 shows the spatial distribution of the resilient modulus of the two CTA base layers under a load of 1006 kPa after considering stress dependence. The vehicle load clearly changed the modulus field inside the base layer. The resilient modulus of both structures was no longer uniformly distributed. Instead, a clear high-modulus zone formed near the load center. As the horizontal distance from the load center and the depth increased, the modulus of the CTA base layer gradually decreased. The contours spread outward in an arc shape from the loading area, indicating that the spatial variation in modulus was closely related to the stress diffusion induced by the vehicle load. As shown in Figure 7a, the OPC-CTA base layer exhibited a higher modulus near the load center, with a maximum predicted modulus of approximately 3.69 × 10^3^ MPa. The high-modulus zone was mainly concentrated at the top of the base layer and near the load center. The modulus decreased rapidly with increasing horizontal distance. For example, near the top of the base layer, the modulus decreased from more than 3000 MPa near the load center to less than 2000 MPa away from the loading area. At greater depths, the modulus further approached the reference modulus. The contours near the load center were relatively dense, indicating a large modulus gradient in this region. The vertical load places the upper part of the base layer in a clear compressive stress state, which increases the stress-dependent resilient modulus.

In comparison, the maximum modulus of the SSC-CTA base layer in Figure 7b was approximately 2.68 × 10^3^ MPa, which was lower than that of the OPC-CTA base layer. However, the load-induced spatial variation in modulus was still clear. The high-modulus zone of the SSC-CTA base layer was also concentrated beneath the load center and gradually decreased along the horizontal and vertical directions. Compared with OPC-CTA, the modulus contours of SSC-CTA changed more smoothly. This indicates that its modulus increase was smaller, although stress dependence still played a role within a certain depth range in the upper base layer. In other words, OPC-CTA showed stronger local modulus enhancement near the load center, while SSC-CTA showed a more gradual spatial transition in modulus.

**Figure 8:**
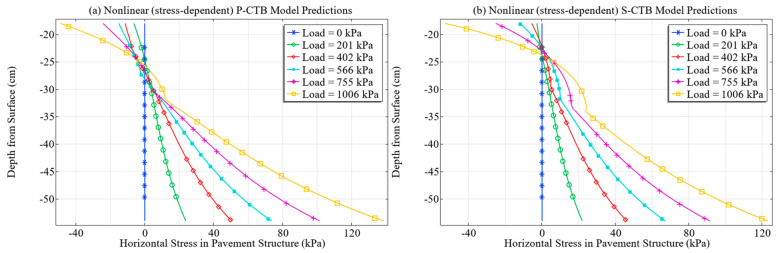
Horizontal stress distribution within the base layer of different pavement structures.

Figure 8 shows the depth distributions of horizontal stress in the OPC-CTA base layer and SSC-CTA base layer under different vehicle load levels after considering stress dependence. As the load level increased from 201 kPa to 1006 kPa, the magnitude of horizontal stress in the base layer increased significantly. The stress curves under different load levels also gradually expanded to both sides, indicating that vehicle loading had a clear effect on the internal stress state of the base layer. Overall, both base layers exhibited a typical stress pattern of compression in the upper part and tension in the lower part. The horizontal stress near the top of the base layer was negative, while it gradually changed from compressive stress to tensile stress with increasing depth. The maximum tensile stress occurred near the bottom of the base layer. Comparison between Figure 8a and Figure 8b shows that the horizontal stress distributions of the OPC-CTA base layer and the SSC-CTA base layer are generally similar. However, clear differences can be observed in the compression–tension transition position and the tensile stress level at the bottom of the base layer. Compared with the OPC-CTA base layer, the tensile zone of the SSC-CTA base layer moved upward. This phenomenon is mainly related to the lower modulus of the SSC-CTA base layer. With the decrease in modulus, the overall stiffness of the base layer decreases. Under external loading, the structure is more likely to undergo bending deformation.

In terms of the maximum horizontal tensile stress at the bottom of the base layer, the SSC-CTA base layer always showed a lower value than the OPC-CTA base layer under the same load level. Specifically, under the loads of 201, 402, 566, 755, and 1006 kPa, the maximum tensile stress at the bottom of the SSC-CTA base layer decreased by 12.31%, 10.88%, 9.62%, 9.00%, and 8.60%, respectively, compared with that of the OPC-CTA base layer. This indicates that the SSC-CTA base layer has certain advantages in controlling bottom tensile stress, although its modulus is lower.

For the unfavorable stress state with high horizontal tensile stress at the bottom of the subbase, Equation (14) was further used to predict the remaining fatigue life at the bottom of the subbase under different load levels. The results are listed in Table 8. As shown in the table, when the vehicle load increased from 201 kPa to 1006 kPa, the predicted fatigue life at the bottom of the subbase decreased clearly for both pavement structures. For the OPC-CTA structure, the remaining fatigue life decreased from 80,680 cycles at 201 kPa to 6983 cycles at 1006 kPa. For the SSC-CTA structure, it decreased from 84,299 cycles to 9490 cycles. Both structures showed a rapid reduction in fatigue life with increasing load. This suggests that, under high loading, the tensile stress at the bottom of the subbase increases significantly. As a result, fatigue damage accumulates faster, and the number of fatigue loading cycles that the structure can sustain is greatly reduced.

In terms of material comparison, the predicted fatigue life of the SSC-CTA structure was always higher than that of the OPC-CTA structure under the same load level. Specifically, under the loads of 201, 402, 566, 755, and 1006 kPa, the remaining fatigue life of the SSC-CTA subbase increased by 4.49%, 14.77%, 22.43%, 29.43%, and 35.90%, respectively, compared with that of the OPC-CTA structure. At lower load levels, the difference in tensile stress at the bottom of the subbase between the two structures was relatively small, so the improvement in fatigue life was limited. At medium and high load levels, the bottom tensile stress caused by vehicle loading increased further. The effects of material properties and structural modulus differences on stress distribution and fatigue life were also amplified. Therefore, the fatigue life improvement of the SSC-CTA structure became more significant. When the equivalent load was approximately 0.7 MPa, the remaining fatigue life of the SSC-CTA subbase increased by 29.43% compared with that of the OPC-CTA structure. This indicates that the SSC-CTA structure already showed a clear fatigue resistance advantage under common loading conditions. When the load further increased to 1006 kPa, the improvement in fatigue life reached 35.90%, suggesting that the SSC-CTA structure had a more pronounced effect on delaying fatigue failure of the subbase under heavy traffic loading.

Table 9 presents the sensitivity of the predicted fatigue life of SSC-CTA to the conversion multiplier of the stress-dependent modulus parameter *k*_3_. When *k*_3_ increased from 18,868 to 22,012, corresponding to an increase in the modulus conversion multiplier from 3.0 to 3.5, the predicted fatigue life showed only minor changes under all loading levels. The variation rate ranged from 0.73% to 3.06%, indicating that the fatigue life prediction was not highly sensitive to this parameter within the examined range. This limited sensitivity can be explained by the role of the stress-dependent modulus in the pavement structure. Increasing *k*_3_ mainly affects the modulus distribution in the upper part of the CTA base layer, where the compressive stress state is more pronounced. Therefore, the primary influence of the conversion multiplier is reflected in the redistribution of the internal stress field. However, fatigue life is mainly governed by the tensile response at the bottom of the lower CTA layer. In this region, the material is primarily under tension, and the modulus used in the fatigue calculation remains close to the minimum modulus value. As a result, increasing the stress-dependent modulus conversion multiplier produces only a limited change in the predicted fatigue life.

## 4. Conclusions

The stress nonlinearity, fatigue damage behavior, and structural response of SSC-CTA were investigated in this study. The differences between SSC-CTA and OPC-CTA in material properties and structural fatigue life were compared through dynamic triaxial tests, indirect tensile fatigue tests, and finite element analysis. Based on the experimental and numerical results, the following conclusions can be drawn.

1. SSC-CTA showed a lower dynamic triaxial resilient modulus than OPC-CTA. The average resilient modulus of SSC-CTA was 978 MPa, which was 15.47% lower than that of OPC-CTA. For both materials, the resilient modulus increased with bulk stress and decreased with octahedral shear stress. The NCHRP 28A model provided accurate predictions of the stress-dependent resilient modulus, with R^2^ values of 0.99 for both materials.
2. Under the same loading mode, SSC-CTA showed lower permanent deformation and higher recoverable deformation than OPC-CTA. This is because SSC can compensate for shrinkage during hydration, which greatly reduces the initial cracks caused by early drying shrinkage and thermal shrinkage. As a result, SSC-CTA exhibits better cracking resistance.
3. SSC-CTA showed better tensile and fatigue resistance than OPC-CTA. The indirect tensile strength of SSC-CTA reached 864.3 kPa, which was 52.65% higher than that of OPC-CTA. The Paris’ law parameters of SSC-CTA were A = 1.07 × 10^−5^ and n = 0.627, both lower than those of OPC-CTA. Although SSC-CTA was subjected to a higher fatigue stress level, its damage growth rate during crack propagation was lower, indicating improved fatigue cracking resistance.
4. The finite element results showed that the SSC-CTA structure reduced the bottom tensile stress of the base layer by 12.31%, 10.88%, 9.62%, 9.00%, and 8.60% under the five loading levels, respectively. The predicted fatigue life of the SSC-CTA structure increased by 4.49–35.90% compared with the OPC-CTA structure. The improvement became more significant as the applied load increased, indicating that SSC-CTA has greater fatigue resistance under medium and heavy traffic loading.

The proposed framework links laboratory material parameters with structural fatigue response by incorporating the stress-dependent resilient modulus and fatigue damage parameters into the finite element model. However, the material parameters were obtained from laboratory specimens cured for 28 days under one moisture and temperature condition. Therefore, the effects of longer curing ages, moisture variation, temperature, sulfate exposure, carbonation, and freeze–thaw cycles were not considered.

## Figures and Tables

**Table 1:** Chemical composition of cementitious materials.

Chemical Composition	Unit	P.O.42.5	Granulated Blast-Furnace Slag	Desulfurized Gypsum
SiO_2_	%	22.31	31.24	2.88
Al_2_O_3_	%	9.76	17.63	1.07
CaO	%	54.36	32.93	37.95
Fe_2_O_3_	%	3.13	0.639	0.384
K_2_O	%	1.03	0.376	0.163
MgO	%	1.01	12.07	0.889
Na_2_O	%	0.20	0.964	0.096
TiO_2_	%	0.43	0.501	0.041
SO_3_	%	3.16	2.71	55.17

**Table 2:** Designed aggregate gradation of CTA mixtures.

Gradation	Percentage Passing by Mass Through Sieve Size (%)										
Sieve size (mm)	19	16	13	9.5	4.75	2.36	1.18	0.6	0.3	0.15	0.075
Designed gradation	100	90.5	81	65.5	39	26.5	17.5	11.5	7.5	5	3.5

**Table 3:** Dynamic resilient modulus loading sequences.

Sequence	Confining Stress *σ*_3_ (MPa)	Cyclic Stress *σ*_d_ (MPa)	Bulk Stress *θ* (MPa)	Octahedral Shear Stress *τ*_oct_ (MPa)	Numbers
0	0.105	0.210	0.53	0.099	1000
1	0.045	0.117	0.25	0.055	100
2	0.055	0.117	0.28	0.055	100
3	0.065	0.117	0.31	0.055	100
4	0.07	0.140	0.35	0.066	100
5	0.055	0.185	0.35	0.087	100
6	0.045	0.215	0.35	0.101	100
7	0.055	0.265	0.43	0.125	100
8	0.075	0.265	0.49	0.125	100
9	0.095	0.265	0.55	0.125	100
10	0.105	0.285	0.60	0.134	100
11	0.09	0.330	0.60	0.156	100
12	0.08	0.360	0.60	0.170	100
13	0.12	0.339	0.70	0.160	100
14	0.14	0.339	0.76	0.160	100
15	0.16	0.339	0.82	0.160	100
16	0.185	0.395	0.95	0.186	100
17	0.18	0.410	0.95	0.193	100
18	0.17	0.440	0.95	0.207	100

Where *θ* is the first invariant of the principal stress tensor, defined as *θ* = *σ_kk_*; *τ*_oct_ is the octahedral shear stress, defined as $\tau_{\mathrm{oct}}=\sqrt{2J_{2}/3}$; *J*_2_ is the second invariant of the deviatoric stress tensor.

**Table 4:** Parameters of resilient modulus prediction models for CTA.

Models	Parameters	OPC-CTA	SSC-CTA
NCHRP-28A model [29]	*k*_3_	5242	5647
	*k*_4_	0.670	0.392
	*k*_5_	−0.297	−0.150
	*p*	<0.05	<0.05
	R^2^	0.99	0.99
	$\Delta_{\mathrm{cul}}$	20.48%	13.54%
Uzan model [30]	*k*_1_	7308	7552
	*k*_2_	0.475	0.275
	*p*	<0.05	<0.05
	R^2^	0.83	0.82
	$\Delta_{\mathrm{cul}}$	128.61%	83.78%

**Table 5:** Indirect tensile properties of CTA.

Material Type	Indirect Tensile Strength (kPa)	Fatigue Stress (kPa)	Initial Horizontal Resilient Modulus (MPa)	Air Void Content (%)
SSC-CTA	864.3	700	20,875	6.9%
OPC-CTA	566.2	450	11,312	8.9%

**Table 6:** Pavement structure numerical simulation settings.

Layer	Thickness (m)	Constitutive Model	Input Parameters
Asphalt surface layer	0.18	Viscoelastic	$E_{\infty },\nu ,\;E_{i}$ $\rho ,T,\tau_{i}$
CTA base layer	0.18	Nonlinear elastic	$M_{r},E_{m},\nu ,\rho$
CTA subbase layer	0.18	Nonlinear elastic	$M_{r},E_{m},\nu ,\rho$
Subgrade	1.4	Elastic	E,ν,ρ

Where $E_{\infty }$ is the long-term equilibrium modulus, $E_{i}$ is the relaxation modulus component, $\tau_{i}$ is the relaxation time component, ν is Poisson’s ratio, ρ is density, *T* is temperature, *E* is Young’s modulus, *Mr* is the resilient modulus, and *M_r,_*_0_ is the initial resilient modulus.

**Table 7:** Input parameter values for pavement structure numerical simulation.

Asphalt surface $E_{\infty }$= 5 MPa, ν = 0.25, ρ =2500 kg/m^3^, T = 35 °C)											
*i*	1	2	3	4	5	6	7	8	9	10	11
*E_i_* (MPa)	4024	3826	2597	2296	252	311	72	43	5	5	5
*τ_i_* (s)	1 × 10^−5^	1 × 10^−4^	1 × 10^−3^	1 × 10^−2^	0.1	1	10	1 × 10^2^	1 × 10^3^	1 × 10^4^	1 × 10^5^
CTA base layer (ν = 0.25, ρ = 2500 kg/m^3^)											
Materials type				*k*_3_		*k*_4_		*k*_5_		*E_m_* (MPa)	
(1) SSC-CTA				15,726		0.67		−0.29		1000	
(2) OPC-CTA				18,868		0.49		−0.435		1200	
Subgrade soil (E = 69 MPa, ν = 0.4, ρ = 2300 kg/m^3^)											

**Table 8:** Predicted residual fatigue life for different types of base courses.

Loading Level	Fatigue Life of P-CTA	Fatigue Life of S-CTA	Increase Rate
201	80,680	84,299	4.49%
402	28,117	32,269	14.77%
566	16,579	20,298	22.43%
755	10,676	13,818	29.43%
1006	6983	9490	35.90%

**Table 9:** Sensitivity analysis of SSC-CTA fatigue life under different *k*_3_ conversion factors.

Loading Level	*k*_3_ = 18,868	*k*_3_ = 22,012	Variation Rate
201	84,299	82,847	1.72%
402	32,269	32,503	0.73%
566	20,298	20,638	1.68%
755	13,818	14,147	2.38%
1006	9490	9780	3.06%

## Data Availability

The original contributions presented in this study are included in the article. Further inquiries can be directed to the corresponding author.

## Abbreviations

The following abbreviations are used in this manuscript:

OPC-CTA	Ordinary Portland cement-treated aggregate
SSC-CTA	Supersulfated cement-treated aggregate
OMC	Optimum moisture content
MDD	Maximum dry density
